# Effect of Ultrafine Powderization and Solid Dispersion Formation via Hot-Melt Extrusion on Antioxidant, Anti-Inflammatory, and the Human Kv1.3 Channel Inhibitory Activities of *Angelica gigas* Nakai

**DOI:** 10.1155/2020/7846176

**Published:** 2020-09-01

**Authors:** Yunyao Jiang, Jingpei Piao, Nan Liu, Jincai Hou, Jianxun Liu, Weicheng Hu

**Affiliations:** ^1^Institute for Chinese Materia Medica, School of Pharmaceutical Sciences, Tsinghua University, Beijing 100084, China; ^2^College of Life Sciences, Jilin Normal University, Siping 136000, China; ^3^Beijing Increasepharm Safety and Efficacy Co. Ltd., Beijing 102206, China; ^4^Jing-Jin-Ji Joint Innovation Pharmaceutical (Beijing) Co. Ltd., Beijing 100083, China; ^5^Beijing Key Laboratory of TCM Pharmacology, Xiyuan Hospital, China Academy of Chinese Medical Sciences, Beijing 100091, China; ^6^Jiangsu Collaborative Innovation Center of Regional Modern Agriculture & Environmental Protection/Jiangsu Key Laboratory for Eco-Agricultural Biotechnology Around Hongze Lake, Huaiyin Normal University, Huaian 223300, China

## Abstract

*Angelica gigas* Nakai (AGN) was first processed by ultrafine grinding technology and hot-melt extrusion (HME). The potential antioxidant and anti-inflammatory activities of AGN with a different process were compared, and the effect on the human Kv1.3 potassium channel was detected. The process of ultrafine powderization on AGN significantly increased the total phenolic and flavonoid contents, antioxidant activity, and DNA damage protective effect. On the contrary, AGN solid dispersion (AGN-SD) based on Soluplus^®^ showed the highest inhibitory effect on NO production and the human Kv1.3 channel. In addition, AGN-SD inhibited the production of prostaglandin E2 and intracellular reactive oxygen species and the mRNA expression of inducible nitric oxide synthase, cyclooxygenase-2, interleukin 1*β*, and interleukin 6. Taken together, these results suggest that ultrafine powderization and solid dispersion formation via HME can significantly improve the biological activities of AGN. The results also suggested that ultrafine powderization and HME may be developed and applied in the pharmaceutical industry.

## 1. Introduction

Reactive oxygen species (ROS) are chemically oxygen-containing molecules. ROS form as a natural byproduct of the normal metabolism of oxygen and have important roles in cell signaling and homeostasis. The ROS levels could be increased due to environmental pollutants, radiation, chemicals, toxins, food habits, and physical stress. Under these conditions, due to the imbalance between the antioxidants and ROS, the significantly higher level of free radical synthesis will occur. Synthesis of an excess amount of free radicals causes several chronic and degenerative diseases such as aging, coronary heart disease, atherosclerosis, inflammation, stroke, diabetes mellitus, cancer, asthma, arthritis, and other age-related diseases. In addition, free radicals can induce cellular injuries by initiating the peroxidation of polyunsaturated fatty acids in biological membranes [1]. Free radicals are scavenged by the body's natural antioxidant defense systems such as superoxide dismutase, glutathione peroxidase, and catalase. However, endogenous antioxidants are unable to completely remove all free radicals. Therefore, exogenous antioxidants are needed to help the body to decrease the antioxidant stress [2]. Inflammation is a complex biological process and associated with many chronic human diseases including cancer, diabetes, cardiovascular arthritis, autoimmune disorders, and neurodegenerative diseases [3]. Macrophages play a key role in the process of inflammation on account of their functions in innate immunity and adaptive immunity. The activated macrophages by bacterial lipopolysaccharides (LPS) initiate defensive reactions and release inflammatory mediators including nitric oxide (NO) and prostaglandin E2 (PGE2) and proinflammatory cytokines such as tumour necrosis factor-*α* (TNF-*α*), interleukin 1*β* (IL-1*β*), and interleukin 6 (IL-6) to improve the defensive ability [4]. The generation of proinflammatory mediators such as NO and PGE_2_ is induced by inducible nitric oxide synthase (iNOS) and cyclooxygenase-2 (COX-2), which are strongly involved in inflammation [5]. Therefore, the inhibition of the proinflammatory mediators and cytokines is an effective approach to treat diseases with anti-inflammatory components. In the current study, the pharmacological target of the Kv1.3 potassium channels for immunomodulation of autoimmune diseases has been found, and the therapeutic efficacy of some potent peptide Kv1.3 channel blockers for autoimmune diseases including multiple sclerosis, rheumatoid arthritis, type-1 diabetes, and psoriasis in animal models has been reported [6]. The Kv1.3 channels regulate the resting membrane potential and Ca^2+^signaling to interfere the proliferation and activation of effector memory T cells, which play a main part in the pathogenic mechanism of autoimmune diseases [7]. Therefore, the discovery of more Kv1.3 channel blockers makes sense for drug development.

Ultrafine grinding technology is an effective way to make ultrafine powder. Inside the smaller particles, the core materials can readily diffuse to the surface of the particle. Therefore, the release of core materials from particles can be controlled to attain the desired level of the optimal efficacy by regulating the particle characteristics [8]. Concentration is the driving force for almost all drugs to pass through the biological membranes and eventually be absorbed. For this reason, the efficacy of poorly water-soluble drugs can be seriously limited by the poor water solubility [9]. Pharmaceutical hot-melt extrusion (HME), a solventless polymer processing technique, currently is researched to increase solubility and bioavailability of the poorly water-soluble active pharmaceutical ingredients (APIs) in industry and academia [10]. In the HME process, APIs are embedded in the polymer melts in an amorphous state by distributive and dispersive mixing and diffused to form solid dispersions. It has been reported that HME has been applied to produce solid dispersions of botanical medicines [11]. Polyvinyl caprolactam-polyvinyl acetate-polyethylene glycol graft copolymer (Soluplus®), a new thermoplastic polymer, is an amphiphilic molecule and designed and developed for forming solid solutions [12]. In the present study, the ultrafine grinding technology was used to reduce particle size, and HME was used to prepare *Angelica gigas* Nakai solid dispersions (AGN-SD) on a polymeric matrix. The aim of this work was to evaluate the effect of particle size reduction by ultrafine powderization and solid dispersion formation by HME on antioxidant, anti-inflammatory, and the human Kv1.3 channel inhibitory activities of AGN.

## 2. Materials and Methods

### 2.1. Materials and Chemicals

Four different processing of AGN powder were prepared as described previously [13]. Then, these four powders were extracted 3 times with deionized water at room temperature for 3 h. After vacuum filtration, the supernatant was concentrated to afford the water extract of coarse powder (WEC), water extract of ultrafine powder (WEU), water extract of HME powder (WEH), and water extract of AGN-SD (WES). The dried samples were weighed and kept in a refrigerator until further analysis. 2,2-Diphenyl-1-picrylhydrazyl (DPPH), butylated hydroxyanisole (BHA), 2-deoxy-D-ribose, ascorbic acid, *α*-tocopherol, ethylenediaminetetraacetic acid (EDTA), 3-(2-pyridyl)-5,6-bis (4-phenyl-sulfonic acid)-1,2,4-triazine (ferrozine), tannic acid, quercetin, trichloroacetic acid (TCA), aluminium chloride hexahydrate (AlCl_3_·6H_2_O), Folin–Ciocalteu reagent, nitro blue tetrazolium (NBT), ferric chloride, phenazine methosulfate (PMS), *β*-nicotinamide adenine dinucleotide (NADH), 1-(4,5-dimethylthiazol-2-yl)-3,5-diphenylformazan (MTT), and LPS (*E. coli* 0111:B4) were purchased from Sigma (St. Louis, MO, USA). RPMI medium 1640 and fetal bovine serum (FBS) were acquired from Gibco BRL (Grand Island, NY, USA). All culture supplies were obtained from BD Falcon (BD, Franklin Lakes, NJ, USA).

### 2.2. Determination of Total Phenolic and Flavonoid Contents

Total phenolic content in the obtained four different extracts was determined by the Folin–Ciocalteu method [14] with minor modifications. In brief, 0.1 mL of the sample or different concentrations of tannic acid were mixed with 500 *µ*L of 10% Folin–Ciocalteu reagent. Then, the reaction was initiated by adding 400 *µ*L of sodium carbonate solution (7.5%, w/v) for 30 min at room temperature, and the absorbance of the mixture was measured at 765 nm against the blank. Total phenolic content was expressed as mg tannic acid equivalents/g using the equation based on the calibration curve.

To estimate the total flavonoid content, 500 *µ*L of the sample (1 mg/mL) was mixed with 100 *µ*L of 10% AlCl_3_·6H_2_O, 100 *µ*L of 1 M potassium acetate, 2.8 mL of deionized water, and 1.5 mL 95% ethanol. After incubation at room temperature for 40 min, the absorbance of the mixture was measured at 415 nm against the blank. Quercetin was chosen as the standard, and the data were then converted into mg quercetin equivalents/g.

### 2.3. Reducing Power Assay

The reducing power ability of different samples was determined according to the method described by Singh and Rajini [15] with some modifications. In brief, the reaction mixture consisted of 1 mL of the sample, 2.5 mL of 0.2 M sodium phosphate buffer (pH 6.6), and 2.5 mL of 0.1% potassium ferricyanide. The reaction mixture was incubated at 50°C for 30 min. After incubation, a volume of 2.5 mL of TCA solution (10%) was added to the mixture, which was then centrifuged at 3000 rpm for 10 min. The upper layer of the mixture (2.5 mL) was mixed with an equal volume of distilled water and 0.5 mL of 0.1% ferric chloride solution. The absorbance was determined at 700 nm against the blank. Ascorbic acid was used as a positive control.

### 2.4. Superoxide Radical Scavenging Assay

Superoxide radical scavenging activity of different samples was measured by the reduction of NBT according to the previously reported method [16] with minor modifications. The reaction mixture contained 1 mL of 0.1 M phosphate buffer (pH 7.4), 100 *μ*L of NADH (468 *μ*M), 100 *μ*L of NBT (150 *μ*M), 20 *μ*L of PMS (60 *μ*M), and100 *μ*L of each sample. After 5 min of incubation at room temperature, the absorbance was measured at 560 nm against the blank. Gallic acid was used as a positive control.

### 2.5. Metal Chelating Assay

The metal chelating activity of different samples was measured using the colorimetric method described previously [17] with a slight modification. In brief, 200 *µ*L of the sample was mixed with 20 *µ*L of 2 mM FeCl_2_ in 740 *µ*L of methanol. The reaction was initiated by the addition of 40 *µ*L of 5 mM ferrozine. After 10 min, the absorbance of the solution was determined at 562 nm against the blank. BHA and EDTA were used as positive controls.

### 2.6. DNA Damage Protection Assay

The DNA damage protective ability of different samples was analyzed as described in a previous report [18]. The reaction mixture included 4 *μ*L of DNA isolated from RAW 264.7 cells, 3 *μ*L of 50 mM phosphate buffer (pH 7.4), Fenton's reagent (3 *μ*L of 1 mM FeSO4 and 4 *μ*L of 0.1 mM H_2_O_2_), and 10 *μ*L of each sample. The reaction mixture was incubated at 37°C for 30 min. After incubation, the reaction was terminated by adding 5 *μ*L of gallic acid. DNA damage was analyzed by 1% agarose gel electrophoresis stained with ethidium bromide followed by visualization under UV light using a Gel Doc XR system (Bio-Rad, Hercules, CA, USA). The optical density of each DNA band was recorded using Quantity One software (Bio-Rad, Hercules, CA, USA).

### 2.7. Cell Line and Cell Culture

The RAW 264.7 mouse macrophage cell line was provided by American Type Culture Collection (ATCC) (Manassas, VA, USA). RAW 264.7 cells were plated into T-75 flasks and cultured in RPMI 1640 Medium containing 10% heat-inactivated FBS and 1% penicillin/streptomycin at 37°C in a humidified atmosphere of 95% air and 5% CO_2_.

### 2.8. Cell Viability Assay

The cytotoxicity of samples on RAW 264.7 cells was investigated using MTT assay. Cells were seeded into 96-well plates at a density of 1 × 10^5^ cells/well. After incubation for 18 h, cells were exposed to the medium along with samples at different concentrations for 24 h. After that, the medium was removed carefully, and 10 *µ*L of MTT solution (5 mg/mL in phosphate-buffered saline) and 90 *µ*L of FBS-free medium were added to each well and incubated for 4 h at 37°C. After incubation, the colored formazan crystals were solubilized with 200 *μ*L of DMSO and quantified by measuring absorbance at 490 nm using an enzyme-linked immunosorbent assay (ELISA) plate reader. The data are expressed as the percentage of the control optical density (OD) values for each experiment.

### 2.9. Quantification of NO and PGE2 Production in LPS-Induced RAW 264.7 Cells

RAW 264.7 cells (1 × 10^5^ cells/well) were plated in 96-well cell plates and incubated for 18 h. The medium was removed, and the cells were further incubated for 30 min in the absence or presence of various concentrations of samples. Then, they were stimulated with LPS (1 *µ*g/mL) for an additional 24 h. Next, aliquots of 100 *μ*L of the cell culture medium were mixed with 100 *μ*L of Griess reagent (1% sulfanilamide in 5% phosphoric acid and 0.1% naphthylethylenediamine dihydrochloride). After 5 min incubation at room temperature, the absorbance of the samples at 550 nm was measured using an ELISA plate reader. The level of PGE2 was determined using the commercially available kits (Enzo Life Sciences, Farmingdale, NY) according to the manufacturer's instructions.

### 2.10. Reverse Transcription-Polymerase Chain Reaction (RT-PCR) Analysis

RAW 264.7 cells were seeded at a density of 5 × 10^6^ cells/well in a 6-well plate overnight. The cells were pretreated with various concentrations of the sample for 30 min before incubation with LPS (2 *μ*g/mL) for 24 h. Total RNA of the cells was isolated with a TRIzol RNA isolation kit (Invitrogen, Carlsbad, CA, USA). The total RNA was reverse-transcribed to cDNA and used as the template for PCR amplification for COX-2, iNOS, TNF-*α*, IL-1*β,* IL-6, and GAPDH: 5′-CACTACATCCTGACCCACTT-3′ and 5′-ATGCTCCTGCTTGAGTATGT-3′ for COX-2, 5′-CCCTTCCGAAGTTTCTGGCAGCAG-3′ and 5′-GGCTGTCAGAGCCTCGTGGCTTTGG-3′ for iNOS, 5′-TTGACCTCAGCGCTGAGTTG-3′ and 5′-CCTGTAGCCCACGTCGTAGC-3′ for TNF-*α*, 5′-TGGACGGACCCCAAAAGATG-3′ and 5′-AGAAGGTGCTCATGTCCTCA-3′ for IL-1*β*, 5′-GTTCTCTGGGAAATCGTGGA-3′ and 5′-TGTACTCCAGGTAGCTATGG-3′ for IL-6, and 5′-CACTCACGGCAAATTCAACGGCA-3′ and 5′-GACTCCACGACATACTCAGCAC-3′ for GAPDH. The amplified PCR products were analyzed by 1% agarose gel electrophoresis stained with ethidium bromide followed by visualization under UV light using a Gel Doc XR system (Bio-Rad, Hercules, CA, USA).

### 2.11. Determination of Intracellular ROS

RAW 264.7 cells were plated in 6-well plates at a density of 1 × 10^6^ cells/well with 2 mL culture medium for 18 h and exposed to the sample with different concentrations for 30 min before exposure to 2 *μ*g/mL LPS. After incubation for 24 h, cells were treated with DCFH-DA (20 *μ*M) for 30 min in the dark. Then, cells were washed thrice with PBS at 37°C and detached by scraping them with a sterile cell scraper. Intracellular ROS was measured by flow cytometry (Becton-Dickinson, Franklin Lakes, NJ, USA) at an excitation wavelength of 485 nm and an emission wavelength of 535 nm.

### 2.12. Expression of Kv1.3 in Oocytes

cRNA was synthesized by in vitro transcription using message machine T7 kits (Ambion, Austin, TX, USA) and injected into stage V and VI oocytes which were surgically isolated from female *Xenopus laevis* (Nasco, Modesto, CA, USA) anesthetized with 0.17% tricaine methanesulphonate (Sigma, St. Louis, MO, USA). The injected oocytes were maintained in modified Barth`s solution which contained 88 mM NaCl, 1 mM KCl, 0.4 mM CaCl, 0.33 mM Ca(NO_3_)_2_, 1 mM MgSO_4_, 2.4 mM NaHCO_3_, 10 mM HEPES (pH 7.4), and 50 *µ*g/mL gentamicin sulfate at 17°C. The currents were measured three to six days after injection.

### 2.13. Solution and Voltage-Clamp Recording from Oocytes

Normal Ringer's solutions which contained 96 mM NaCl, 2 mM KCl, 1.8 mM CaCl_2_, 1 mM MgCl_2_, and 10 mM HEPES (pH 7.4) were applied to oocytes by continuous perfusion of the chamber, and solution exchanges were completed within 3 min. Currents were measured after 10 min of the solution exchange at room temperature (20°C–23°C) with a two-microelectrode voltage-clamp amplifier (Warner Instruments, Hamden, CT, USA). Electrodes were filled with 3 M KCl and had a resistance of 2–4 MΩ and 1–2 MΩ for voltage-recording electrodes and current-passing electrodes, respectively. Stimulation and data acquisition were controlled with an AD-DA converter (Digital 1200, Axon Instruments) and pCLAMP software (v5.1, Axon Instruments).

### 2.14. Statistical Analyses

All tests were carried out independently in triplicate. Data are expressed as mean ± standard derivation. One-way analysis of variance (ANOVA) was used to determine significant differences between groups followed by Duncan's multiple range test. Differences between groups were considered significant at *p* < 0.05. All analyses were performed using SPSS for Windows 7, version 20 (SPSS Inc., Chicago, IL, USA).

## 3. Results

### 3.1. Total Phenolic and Total Flavonoid Contents

Table 1 shows the total phenolic and total flavonoid contents of water extracts of AGN with a different process. The total phenolic contents in WEC, WEU, WEH, and WES were 25.94, 36.33, 22.05, and 17.99 mg tannic acid equivalents/g, respectively. Among the four extracts, WEU contained the highest concentration of flavonoids (6.77 mg quercetin equivalents/g), which was followed by WEC (5.55 mg quercetin equivalents/g), WEH (5.37 mg quercetin equivalents/g), and WES (4.74 mg quercetin equivalents/g).

### 3.2. Antioxidant Activity

**Figure 1:**
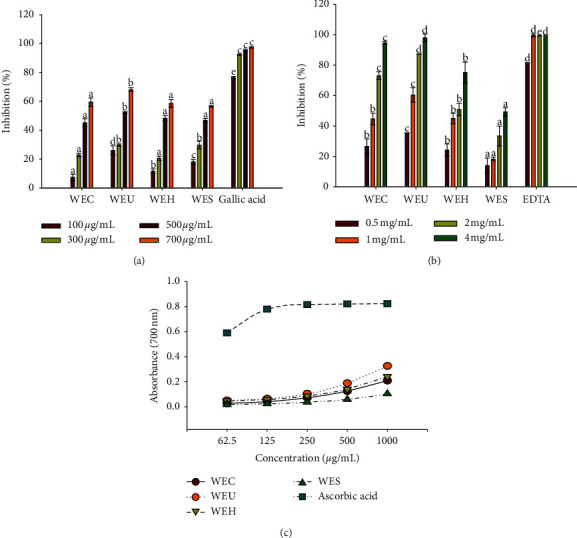
Antioxidant activity of water extracts of different processed AGN. (a) Superoxide radical scavenging activity. (b) Metal chelating activity. (c) Reducing power ability. Each value is expressed as the mean ± SD (*n* = 3). Values at the same concentration are significantly different by Duncan's multiple range test (*p* < 0.05).

WEU showed the significantly strongest scavenging effect on superoxide radicals compared with other extracts at each concentration (Figure 1(a)). WEU scavenged more than 65% of superoxide radicals when the concentration was 700 *μ*g/mL. However, the superoxide radical scavenging activity of WEU was significantly lower than gallic acid. WEH and WES exhibited a higher inhibition than WEC at a low concentration. The scavenging activity of WEC, WEH, and WES on superoxide radicals was not significantly different.

As shown in Figure 1(b), WES showed the lowest metal chelating activity among all extracts. WEC and WES did not exhibit significantly different effect on chelating ferrous ions at 0.5 and 1 mg/mL; however, the chelating effect of WEC was significantly stronger than that of WEH with increasing concentration. WEU showed the significantly strongest metal chelating activity when compared with other extracts. WEU chelated 98% of ferrous ions at a concentration of 4 mg/mL, which was the same with the inhibition of EDTA.

All the extracts showed concentration-dependent reducing power activity (Figure 1(c)). WEU exhibited significantly stronger reducing power than the remaining extracts at all the tested concentrations, which was followed by WEH, WEC, and WES. Among all concentrations of WEU analyzed, 1000 *μ*g/mL exhibited maximum reducing power than the remaining concentrations. However, reducing power ability of WEU was lower than that of ascorbic acid.

### 3.3. Correlations between Antioxidant Activities and Total Phenolic Content

Table 2 shows the correlation analysis between antioxidant activities and total phenolic content of water extracts of AGN with a different process. In this study, correlation analysis exhibited a good correlation between total phenolic content and superoxide radical scavenging activity (*R*^2^ = 0.9631), metal chelating activity (*R*^2^ = 0.7983), or reducing power activity (*R*^2^ = 0.8965).

### 3.4. DNA Damage Protective Activity

**Figure 2:**
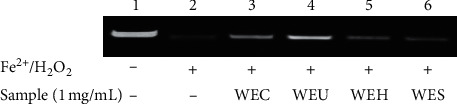
Visualization of the damage induced by hydroxyl radicals on genomic DNA in the presence and absence of water extracts of different processed AGN by agarose gel electrophoresis.

The protective effect of water extracts of AGN with the different process on Fe^2+^/H_2_O_2_-induced DNA damage was present in the electrophoretic pattern. As shown in Figure 2, the DNA treated without Fe^2+^/H_2_O_2_ showed a bright and wide band (lane 1) on agarose gel electrophoresis. The reaction between O^2^•− and H_2_O_2_ can produce OH• in the presence of metal ions. And the OH• induced the oxidative damage of DNA. In this result, DNA was completely degraded (lane 2) by treatment with Fe^2+^ and H_2_O_2_. The DNA damage caused by Fe^2+^/H_2_O_2_ was reduced in the presence of extracts (lanes 3, 4, 5, and 6). Among all extracts, WEU showed the highest DNA damage protective ability.

### 3.5. Anti-Inflammatory Activity

**Figure 3:**
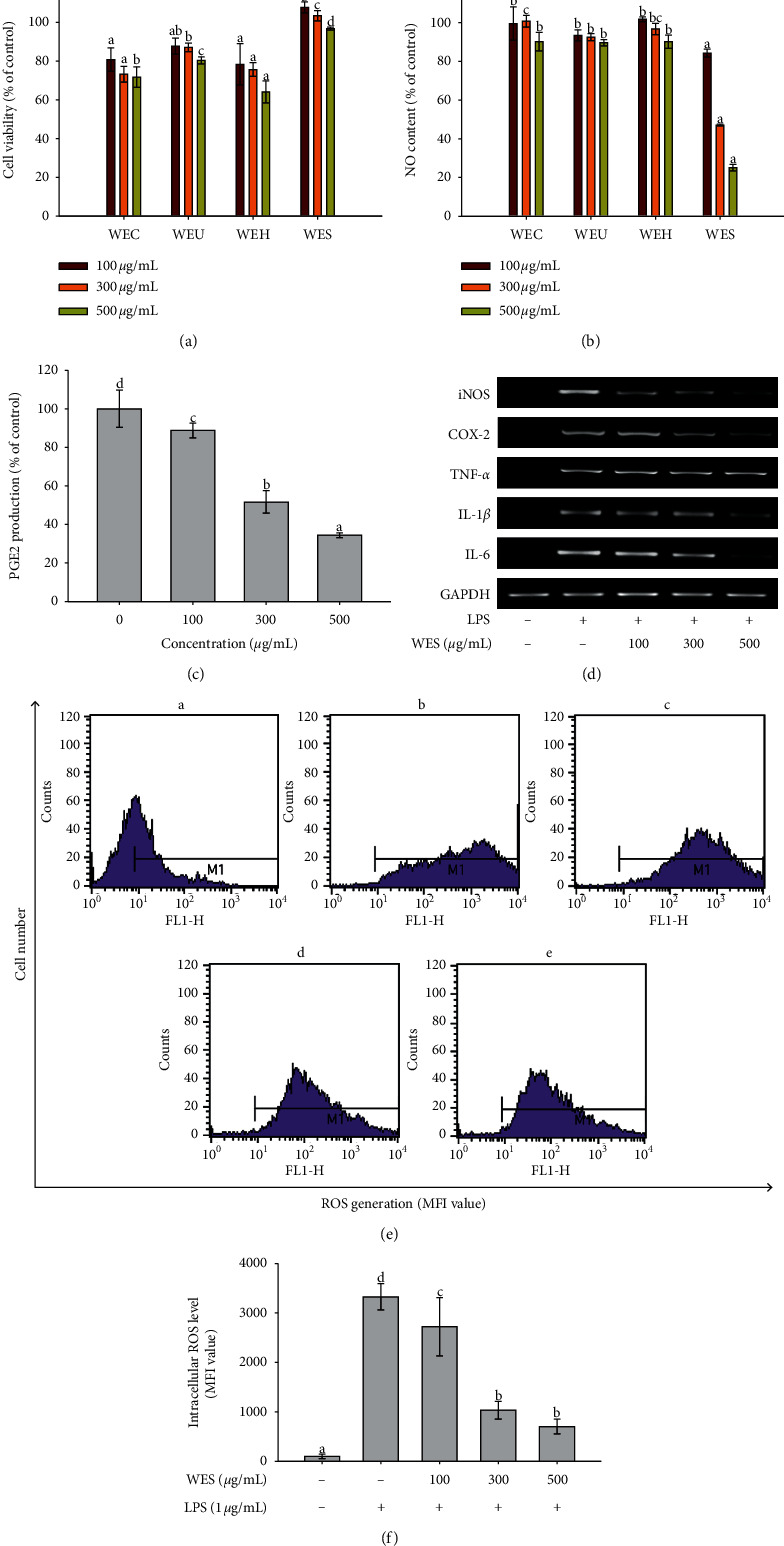
Anti-inflammatory activity of water extracts of different processed AGN. (a) Effect of water extracts of different processed AGN on the cell viability. (b) Inhibitory effect of water extracts of different processed AGN on LPS-induced NO production in RAW 264.7 macrophages. (c) Inhibitory effect of WES on LPS-induced PGE2 production in RAW 264.7 macrophages. (d) Effect of WES on iNOS, COX-2, TNF-*α*, IL-1*β*, and IL-6 expressions in LPS-treated RAW264.7 cells. (e) Suppression of LPS-induced ROS in RAW 264.7 cells in the presence of WES: (A) control (no treatment); (B) 1 *μ*g/mL LPS treatment; (C) 1 *μ*g/mL LPS treatment after 100 *μ*g/mL of WES addition; (D) 1 *μ*g/mL LPS treatment after 300 *μ*g/mL of WES addition; (E) 1 *μ*g/mL LPS treatment after 500 *μ*g/mL of WES addition. (f) Histogram showing intracellular ROS in RAW 264.7 macrophage cells. Each value is expressed as the mean ± SD (*n* = 3). Values in the same column are significantly different by Duncan's multiple range test (*p* < 0.05).

Cell viability was tested at various concentrations of samples by the mitochondrial reduction of MTT and expressed as a percentage of viability with respect to the control group, which was considered as 100% viable. As shown in Figure 3(a), the viability of RAW 264.7 cells treated with WES was significantly higher than the remaining extracts at all the tested concentrations. The cell viability was more than 95% when the cells were treated with WES. The generation of NO in LPS-simulated RAW 264.7 cells was measured by the Griess reaction. NO content in RAW 264.7 cells treated with WES was the significantly lowest compared with other extracts at each concentration (Figure 3(b)). WES inhibited the NO production significantly by 15.79%, 52.87%, and 74.88% at a concentration of 100, 300, and 500 *μ*g/mL, respectively. The results showed that WES inhibited the NO production in a concentration-dependent manner in LPS-stimulated RAW 264.7 cells.

As shown in Figure 3(c), WES showed a dose-dependent inhibitory effect on PGE2 production in LPS-simulated RAW 264.7 cells. The production of PGE2 was significantly decreased by WES with increasing concentration. WES inhibited 65.62% PGE2 production at a concentration of 500 *μ*g/mL. RT-PCR was performed to evaluate the inhibitory activity of WES on iNOS, COX-2, IL-1*β*, and IL-6 mRNA expressions in LPS-stimulated RAW264.7 cells. As shown in Figure 3(d), iNOS, COX-2, TNF-*α*, IL-1*β*, and IL-6 expressions were increased after treatment with LPS. WES markedly inhibited the expression of iNOS, COX-2, IL-1*β*, and IL-6 with increasing concentrations in LPS-stimulated RAW 264.7 cells. WES decreased iNOS, COX-2, IL-1*β*, and IL-6 expression to the nearly same levels with the treatment without LPS at a concentration of 500 *μ*g/mL. WES did not affect the house-keeping gene expression, and mRNA levels of TNF-*α* were not inhibited under LPS-stimulated RAW 264.7 cells.

ROS level in LPS-induced RAW 264.7 cells treated with WES was measured with the fluorescence probe DCFH-DA by the flow cytometry system. As shown in Figures 3(e) and 3(f), low ROS level was found in the control cells. However, ROS level was significantly increased when cells were treated with LPS. LPS obviously stimulated ROS generation. WES significantly inhibited ROS generation in LPS-induced RAW 264.7 cells. The ROS level was reduced by WES with increasing concentration, and ROS level was the lowest at 500 *μ*g/mL.

### 3.6. Inhibition Effect on the Human Kv1.3 Channel

**Figure 4:**
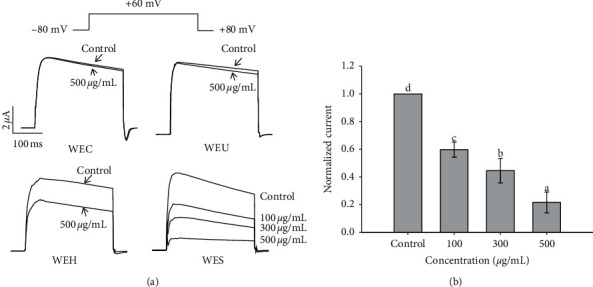
The inhibitory effect of water extracts of different processed AGN on the human Kv1.3 channel currents. (a) Current traces were evoked by 1 s depolarization to +60 mV from a holding potential of −80 mV under the control condition and exposure to water extracts of different processed AGN. (b) Plot of normalized currents measured at their peak under the control condition and exposure to various concentrations of WES. Each control current was normalized to 1 (*n* = 4). Values in the same column are significantly different by Duncan's multiple range test (*p* < 0.05).

The inhibition effect of water extracts of AGN with the different process on the human Kv1.3 channel current is presented in Figure 4(a). The result of 1 sec depolarization to +60 mV from a holding potential of −80 mV under control condition and exposure to the extract was expressed as the superimposed Kv1.3 current traces. Under the control condition, the Kv1.3 currents were promptly activated and reached a peak value. And then, the Kv1.3 currents were slowly inactivated with the maintenance of the depolarizing pulse. In the presence of WEC or WEU, the peak amplitude of the current was reduced; however, the time course for current decay was not influenced. WEH and WES obviously decreased the peak amplitude of the current and influenced the time course for current decay. 100, 300, and 500 *μ*g/mL WES decreased peak currents by 40.2% ± 5.5%, 55.4% ± 8.9%, and 78.3 ± 7.5%, respectively (Figure 4(b)).

## 4. Discussion

Phenolic compounds and flavonoids are widely distributed in plants, vegetables, and fruits and have been recognized as powerful *in vitro* antioxidants due to their ability to donate hydrogen or electrons, and they form stable radical intermediates [19]. In the present study, WEU contained the highest total phenolic and flavonoid contents, which was followed by WEC, WEH, and WES. The particle size of the ultrafine powder was significantly smaller than that of the coarse powder. The results suggested that reduction in particle size by ultrafine grinding technology could increase the total phenolic and flavonoid contents in the water extract of AGN. Ultrafine grinding technology is a technology, which is a useful tool for making ultrafine powder with good surface properties (dispersibility and solubility), fluidity, water holding capacity, and solubility; also, it can increase the solubility and absorption of the nutritive components [20]. After ultrafine grinding, the fiber matrix was changed or damaged, which caused some phenolic compounds linked or embedded in the matrix to be released or exposed [21]. Although the particle size of AGN was also significantly reduced after HME, the total phenolic and flavonoid contents in WEH or WES were significantly lower than WEC. The reason might be that HME destroyed the total phenolic and flavonoid contents in AGN or decreased the extraction rate. HME technology is more useful from the perspective of lower nutrient losses than other thermal processing techniques. Several HME variables can influence the composition of the finish products. These include raw material characteristics, mixing and conditioning of raw material, barrel temperature, pressure, screw speed, and moisture content. The complex conditions often have the ability to produce negative influences on the bioactive compounds of the HME products. For example, the polyphenol content and antioxidant activity of common bean were significantly decreased after HME processing [22]. Similarly, during HME of the bean/corn mixture, a significant decrease in the total polyphenols and antioxidant activity was observed [23]. Both feed moisture content (12–18%) and barrel temperature (150°C–175°C) cause significant decrease in the total phenolic content [24]. Also, the high barrel temperature and high moisture content may cause phenolic compound decarboxylation and then may promote polymerization of phenols and tannins, leading to reduced extractability and antioxidant activity [25, 26].

Large quantities of superoxide radicals are generated in the body in a variety of metabolic and physiological processes [27]. It has been known that superoxide radicals are the precursor of single oxygen and hydroxyl radicals and very harmful to some cellular components [28]. The superoxide radical scavenging ability of extracts was determined by a PMS-NADH superoxide generating system and compared with gallic acid. The transition metal ions can catalyze the production of ROS such as hydroxyl radical and superoxide radical, which induce the oxidation of unsaturated lipids and promote the oxidative damage [29]. Thus, metal chelating activity is one of the criteria to evaluate the antioxidant activity of antioxidants. In addition, the reducing power also gives estimable information on the potential that the tested sample can be used as an antioxidant agent because the antioxidants serve on reducing agents and are oxidized to end the ongoing oxidation reactions in the systems in the body [30]. Among all the tested samples, WEU showed the best effect on all the antioxidant assays including superoxide radical scavenging assay, metal chelating assay, and reducing power assay. In this study, the total phenolic content showed good correlation with antioxidant activities. This suggested that the total phenolic content played a decisive role in the antioxidant activities of water extracts of AGN. The stability of genome and the normal life cycle of the cells are affected by DNA damage which has been connected with cell cycle regulation, repair pathways, and cell death mechanisms [31]. DNA integrity can be affected by a combination of factors, including spontaneous cell processes such as ROS production. Therefore, DNA damage protective activity is usually utilized to characterize antioxidant efficacy. In the DNA damage protection assay, WEU also showed the strongest protective capability against DNA damage followed by WEC, WEH, and WES. The ranking was the same with that of the total phenolic content.

NO, which is a short-lived free radical, plays a role in mediating many biological functions such as vascular homeostasis, neurotransmission, antimicrobial defense, and antitumor activities [32]. However, it has been demonstrated that increased NO production was associated with the pathogenesis of inflammatory tissue injury and several additional disease states. Our present research used MTT assays to determine cytotoxic effects of four samples on RAW 264.7 cells. The NO production inhibitory effect of WES had no relationship with the cytotoxicity as assessed by the MTT assay. In view of the best NO inhibitory effect and the characteristic of noncytotoxicity, WES was selected for future studies. PGE2 has been identified as the primary prostaglandin in the process of acute inflammation and chronic diseases such as rheumatoid arthritis and inflammatory bowel disease [33]. Therefore, the interference of PGE2 generation in pharmacology is considered to be a pathway to alleviate a lot of disease states which are mediated by macrophage activation [34]. In our research, WES effectively inhibited PGE2 production in LPS-stimulated RAW 264.7 cells. iNOS is a product of the transcription expression of activated macrophages and is the reason for the prolonged and profound production of NO [35]. COX-2 is an inducible enzyme, and overexpression of it has been deemed to involve in the pathogenesis of various inflammatory diseases, angiogenesis, and cancer cell invasion [36]. The expression of iNOS and COX-2 induces the generation of excessive amounts of NO and PGE2 [37]. In the present study, mRNA expression of iNOS and COX-2 was attenuated by WES treatment, which might indicate that WES inhibited the production of NO and PGE2 by downregulating the expression level of iNOS and COX-2. As proinflammatory cytokines, TNF-*α*, IL-6, and IL-1*β* contribute to the pathogenesis of some inflammation-related diseases. IL-6 is a pivotal cytokine in defense against inflammation, and dysregulation of IL-6 production has been associated with the development of a variety of autoimmune inflammatory diseases [38]. It has been confirmed that IL-1*β* can increase the expression of nearly all other cytokines [39].

In this study, we found that WES attenuated the mRNA expression of IL-6 and IL-1*β*. However, WES did not exhibit a dramatic effect on inhibiting TNF-*α* expression. The excess intracellular ROS can increase inflammation by activating the ROS-sensitive signaling pathways and downstream transcriptional factors and ultimately increasing the genetic expression of inflammation [40]. Our results demonstrated that WES played an anti-inflammatory role to inhibit the ROS production in LPS-stimulated macrophage cells.

In the previous studies, Kv1.3 blocker peptide toxins obviously showed the activity to restrain T-cell-mediated inflammatory reactions [41]. On the basis of these studies, Kv1.3 blockers may have therapeutic efficacy to inflammatory and autoimmune diseases [42]. In our study, water extracts of AGN acted on a Kv1.3 blocker to inhibit the Kv1.3 channels, which may be the fundamental reason that AGN showed the anti-inflammatory activity. The correlation of the Kv1.3 channels and inflammation-related signaling pathways needs to be further researched.

## 5. Conclusions

We demonstrated that the process of ultrafine powderization on AGN effectively improved the antioxidant activity of AGN by elevating the total phenolic and total flavonoid contents and increasing the superoxide radical scavenging, metal chelating, reducing power, and DNA damage protective activities. In addition, the formation of solid dispersions of AGN based on Soluplus® via HME significantly improved the anti-inflammatory activity by inhibiting the production of NO, PGE2, and intracellular ROS and downregulating the gene expression of iNOS, COX-2, and proinflammatory cytokines including IL-6 and IL-1*β*. Solid dispersions of AGN based on Soluplus® also exhibited a better inhibitory effect on the human Kv1.3 channel. The present work suggested that ultrafine powderization and solid dispersion formation via HME are applicable ways to develop a new AGN product which can maximize the pharmacological effect. Also, it encouraged the further development and application in ultrafine powderization and HME process.

## Figures and Tables

**Table 1:** Total phenolic and flavonoid contents of different processed *A. gigas* Nakai.

Samples	Phenolic content (mg tannic acid equivalents/g)	Flavonoid content (mg quercetin equivalents/g)
WEC	25.94 ± 0.35^c^	5.55 ± 0.16^b^
WEU	36.33 ± 0.42^d^	6.77 ± 0.21^c^
WEH	22.05 ± 0.29^b^	5.37 ± 0.21^b^
WES	17.99 ± 0.10^a^	4.74 ± 0.55^a^

Each value is expressed as the mean ± SD (*n* = 3); different letters of the upper index in the same column are significantly different by Duncan's multiple range test (*p* < 0.05).

**Table 2:** Correlations between antioxidant activities and total phenolic content.

Correlations	Total phenolic content (TPC)
Superoxide radical scavenging activity (SRSA)	SRSA = 0.6333TPC + 44.477 (*R*^2^ = 0.9631)
Metal chelating activity (MCA)	MCA = 0.0104TPC − 0.0457 (*R*^2^ = 0.7983)
Reducing power activity (RPA)	RPA = 2.8668TPC − 12.214 (*R*^2^ = 0.8965)

## Data Availability

The data used to support the findings of this study are available from the corresponding author upon request.
